# Neural parameter calibration for large-scale multiagent models

**DOI:** 10.1073/pnas.2216415120

**Published:** 2023-02-10

**Authors:** Thomas Gaskin, Grigorios A. Pavliotis, Mark Girolami

**Affiliations:** ^a^Department of Applied Mathematics and Theoretical Physics, University of Cambridge, Cambridge CB3 0WA, UK; ^b^Department of Mathematics, Imperial College London, London SW7 2AZ, UK; ^c^Department of Engineering, University of Cambridge, Cambridge CB2 1PZ, UK; ^d^The Alan Turing Institute, London NW1 2DB, UK

**Keywords:** multiagent systems, neural differential equations, model calibration, parameter density estimation

## Abstract

Computational models have become a powerful tool in the quantitative sciences to understand the behavior of complex systems that evolve in time. However, they often contain a potentially large number of free parameters whose values cannot be obtained from theory but need to be inferred from data. This is especially the case for models in the social sciences, economics, or computational epidemiology. Yet, many current parameter estimation methods are mathematically involved and computationally slow to run. In this paper, we present a computationally simple and fast method to retrieve accurate probability densities for model parameters using neural differential equations. We present a pipeline comprising multiagent models acting as forward solvers for systems of ordinary or stochastic differential equations and a neural network to then extract parameters from the data generated by the model. The two combined create a powerful tool that can quickly estimate densities on model parameters, even for very large systems. We demonstrate the method on synthetic time series data of the SIR model of the spread of infection and perform an in-depth analysis of the Harris–Wilson model of economic activity on a network, representing a nonconvex problem. For the latter, we apply our method both to synthetic data and to data of economic activity across Greater London. We find that our method calibrates the model orders of magnitude more accurately than a previous study of the same dataset using classical techniques, while running between 195 and 390 times faster.

## Significance

In this work, we consider multiagent models, widely used across the quantitative sciences to analyze complex systems. These often contain parameters which must be estimated from data. While many methods to do so have been developed, they can be mathematically involved or computationally expensive. We present an alternative using neural networks that addresses both these issues. Our method can make accurate predictions from various kinds of data in seconds where more classical techniques, such as MCMC, take hours, thereby presenting researchers across the quantitative disciplines with a valuable tool to estimate relevant parameters and produce more meaningful simulations at a greatly reduced computational cost.

We live in an age of complexity. Thanks to an array of sophisticated and potent computational resources, paired with vast and fruitful reservoirs of data, researchers can increasingly see social, economic, biological, or epidemiological processes as the complex, self-organizing, and dynamical systems they are. A line of great current interest in the mathematical sciences is to calibrate parameters of mathematical models using data, thereby creating computationally efficient, theoretically grounded, and socially beneficial predictive tools. For instance, models of the spread of contagion continue to inform much government policy during the ongoing COVID-19 pandemic (1–5): predictions are generated, subsequently compared to live data, and the underlying model revised accordingly. Computational models have also been used to understand the dynamics of crime and urban violence (6, 7), pedestrian dynamics (8), synchronized oscillations (9), social network dynamics (10–12), chemotaxis and flocking (13–15), population dynamics (16, 17), systemic risk (18), and the dynamics of economic systems (19, 20).

Two modeling paradigms have established themselves over the years: In the first, a system of coupled differential equations is solved to simulate the behavior of a finite number of interacting particles. This is at times implemented using what are called agent-based models (ABMs): a collection of entities (agents) moving through space and time, interacting with each other and their environment, and adapting their behavior or even learning in the process. In the second approach, a model is realized as a discretized version of equations in continuous time and space (21, 22); this approach opens models up to analysis using mean-field theory, stochastic analysis, statistical physics, and kinetic theory (23–26). A large and sophisticated toolset of statistical methods exists to estimate parameters, interaction kernels, or network structures from data, such as maximum likelihood estimators (27–31), Markov chain Monte Carlo methods based on a Bayesian paradigm (32–35), martingale estimators (36), estimation of active terms in ODE and PDE systems (see ref. 37 for a review), entropy maximization (38), and regression-based learning methods (39, 40). More recently, a promising new method has emerged in the form of artificial neural nets. Neural networks have of course prominently been used as powerful pattern-recognition devices and predictive models (41), but, as they become more and more accessible to the scientific community at large, researchers are beginning to apply their computational capabilities across the mathematical disciplines, including in fields heretofore dominated by more classical methods: Examples include finding solutions of partial differential equations (42, 43) or parameter estimation of multiagent models (44, 45). Neural networks, and especially deep neural networks, are mathematically little understood, and their theoretical underpinnings are sparse and mainly restricted to shallow networks (networks with only one hidden layer) (46, 47). This major drawback notwithstanding, recent results seem to indicate that their computational performance can often outstrip that of other, thus-far more rigorously understood approaches, though the method still lies in its infancy.

This work is a contribution to the general push to better understand the possibilities of neural nets as calibration tools for mathematical models of complex dynamics. We present and investigate a simple yet powerful computational scheme to obtain probability densities for model parameters from data. The method combines classical numerical models with machine learning, and in the following case studies, we recover probability densities from noiseless and noisy, synthetic and real, and time series and steady-state data. The case of a nonconvex problem is also considered. Using the well-known SIR model of contagious diseases, we estimate parameter densities from a time series modeling the diffusion of infection through a population on a two-dimensional (2D) domain. In a second study, we use the Harris–Wilson model of economic activity on a network (19) to learn parameter densities both from synthetic steady-state data as well as a real dataset of activity across Greater London. In doing so, we revisit an earlier study of the same dataset that used Bayesian methods to estimate parameters (20). Our proposed method can find estimates for model parameters in seconds, even for large systems, and provides parameter densities for the London dataset in nearly 1 min where the Bayesian approach took between 3 and 7 h. At the same time, the quality of the calibration (in terms of prediction error) is improved by two to three orders of magnitude.

The scope of our approach covers models formulated as coupled differential equations of the kind

[1] $$\begin{array}{c} d\varphi =f(\varphi ;x,t,\lambda )dt, \end{array}$$

or, in the stochastic case,

[2] $$\begin{array}{c} d\varphi =f\left(\varphi ;x,t,\lambda \right)dt+g\left(\varphi ;t\right)dB_{t}, \end{array}$$

where ***φ*** ∈ ℝ^*N*^ is the state vector, **x** is a space-like variable, ***λ*** := (*λ*_1_, ..., *λ*_*p*_)∈ℝ^*p*^ a set of scalar parameters, and **B** an *N*-dimensional stochastic process (such as a Wiener process). (We include **x** to allow for infinite-dimensional problems leading to (stochastic) partial differential equations, though in practice, discretization will often lead to it being absorbed into the state vector.) In a neural ODE or SDE, the scalar parameters ***λ*** are the outputs of a neural network, whose internal parameters are to be learned from data (48). In this work, we investigate the use of such neural ODEs as calibration tools.

In many cases, the spatial topology is given by a network structure, such that the dependency on **x** is more specifically a dependency on a graph adjacency matrix **A**; this is found in many contemporary models of dynamical systems. One might also like to learn network structures from data. By vectorizing **A**, the network may be conceived of as a single vector of parameters ***λ***, transforming the question into one that can indeed be considered within our proposed framework. However, as the scale of this problem is typically of a different order of magnitude, we address it in future work.

The dynamics of systems governed by equations such as Eq. **1** can depend sensitively on the choice of parameters ***λ***. For mathematical models such as those used in the social sciences or computational biology, theoretical estimates for these parameters are often difficult obtain: What is the reproduction number of a novel disease, and how susceptible are different age groups to the disease? What is a good model for the topology of a social network? How should we estimate return on capital rates for different agents in economic models? What are recovery rates for different animal species subject to predator–prey dynamics? Such parameters are difficult to measure and should instead be extracted from data.

## Methodology

### Obtaining Probability Densities from Neural Equations.

**Fig. 1.**
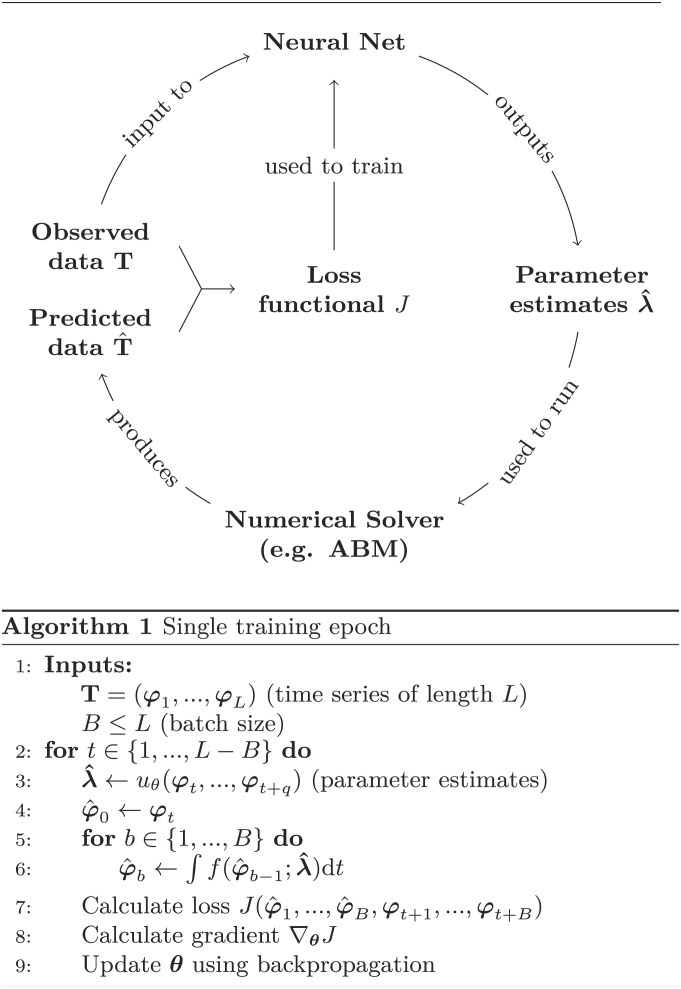
Methodological pipeline proposed in this work. The neural net *u*_*θ*_ takes *q* time series elements as input and outputs parameter predictions. These predictions are fed into a numerical solver, which produces a predicted time series. The true and predicted time series are used to generate a loss functional, which in turn can be used to train the neural net’s internal parameters ***θ***. The goal is to retrieve the true parameters ***λ*** from the data. A single pass over the entire dataset is called an epoch. The dataset is processed in batches, meaning the loss is calculated over *B* steps of the time series before the weights are updated. If *B* = *L*, training is equivalent to batch gradient descent; if *B* = 1, training is equivalent to stochastic gradient descent. The integral in line 6 represents an arbitrary numerical scheme to solve Eq. **1**.

We present a method to estimate parameter densities of ODE or SDE systems by training a neural net to find a set of parameters $\hat{\lambda }$ that, when inserted into the model Eq. **1**, reproduce a given time series **T** = (***φ***_1_, ..., ***φ***_*L*_). A neural network is a function *u*_*θ*_ : ℝ^*N* × *q*^ → ℝ^*p*^, where *q* ≥ 1 represents the number of time series steps that are passed as input (cf. *SI Appendix*, Fig. S1). Its output is the estimated parameters $\hat{\lambda }$, which are used to run a numerical solver for *B* iterations (*B* is the batch size) to produce an estimated time series $\hat{T}\left(\hat{\lambda }\right)=\left({\hat{\varphi }}_{k},...,{\hat{\varphi }}_{k+B}\right)$. This in turn is used to train the internal parameters ***θ*** of the neural net (the weights and biases) via a loss functional $J(\hat{T},T)$. A common choice for *J* is the *l*^2^ norm, which we will use in this work. As $\hat{\lambda }=\hat{\lambda }\left(\theta \right)$, we may calculate the gradient ∇_***θ***_*J* and use it to optimize the internal parameters of the neural net using a backpropagation method of choice; popular choices include stochastic gradient descent, Nesterov schemes, or the Adam optimizer (49, 50). Calculating ∇_***θ***_*J* thus requires differentiating the predicted time series $\hat{T}$, and thereby the system Eq. **1**, with respect to $\hat{\lambda }$; in other words, the loss function contains knowledge of the dynamics of the model. Finally, the true data are once again input to the neural net to produce a new parameter estimate $\hat{\lambda }$, and the cycle starts afresh. A single pass over the entire dataset is called an epoch (cf. Fig. 1). In the following, we will always let *q* = 1. The technicalities of the differentiation procedure are handled by the autodifferentiation scheme, which treats random vectors as constants. For nondifferentiable convex functions (such as ‖ ⋅ ‖), the subgradient of minimum norm is used; see e.g. ref. 51.

This solves an optimization problem, but it does not provide us with any sort of confidence intervals for the predictions. To obtain probability densities, we exploit the fact that as the model trains, it traverses the parameter space ℝ^*p*^, calculating a loss value *J* at each estimate $\hat{\lambda }$. This produces a loss potential $J\left(\hat{\lambda }\right):{\text{ℝ}}^{p}\to \text{ℝ}$, from which a posterior density can be estimated via

[3] $$\begin{array}{c} \pi \left(\hat{\lambda }|T\right)∼\exp\left(-J\left(\hat{T},T\right)\right)\pi^{0}\left(\hat{\lambda }\right), \end{array}$$

with *π*^0^ the prior (34). The marginals are then proportional to

[4] $$\begin{array}{c} \rho \left(\lambda_{i}\right)∼\int \exp\left(-J\right)d{\hat{\lambda }}_{-i}, \end{array}$$

with the subscript −*i* signifying that we are not integrating over ${\hat{\lambda }}_{i}$. In the following, we initialize the neural net’s internal parameters such that the prior is a uniform density. Nonconvexity can be dealt with by training the neural net multiple times on the same dataset, each time with a different initialization, thereby increasing the volume of the sampled parameter space. This comes at a computational cost, but multiple runs can be parallelized and run concurrently, thereby greatly improving performance.

Both the noiseless (Eq. **1**) and the noisy (Eq. **2**) versions of the equations can be used when running the numerical solver (i.e., the forward pass of the pipeline Algorithm 1). Running the solver without noise circumvents having to make any assumptions about the nature of the true underlying noise in the dataset, which will often be unknown. The neural net will then simply fit the best noiseless model to the dataset. However, noise can be added to the solver when randomness is an inherent part of the dynamics and can help make the predictions more robust. Alternatively, the variance of the noise can itself be learned. All these scenarios will be demonstrated in this work.

### Using Neural Equations in Practice.

It is important to reiterate that the neural net is not fitting a dataset in the traditional sense, but rather producing a set of parameters that, when plugged into the governing equations, generate the dataset (or an estimate thereof). Differentiating the loss function (and thereby the physical equations) may seem daunting; but many of the standard machine learning libraries have autodifferentiation features, and so, the differentiation procedure need not (and if possible should not) be implemented manually. In practice, the bottleneck of our method will lie in writing a fast numerical solver that is also compatible with the differentiation procedure of the machine learning package used. Explicitly iterating over agents or network nodes should be avoided and the dynamics instead be implemented as operations on the entire state vector ***φ***. Preimplemented matrix and vector operations should be used as much as possible, as these typically 1) use efficient, tested, and precompiled algorithms and 2) are compatible with autodifferentiation features. We have implemented an open-source code package, written such that it can be easily extended and adapted to further models. See the *SI Appendix* and the README files in the repository.

Aside from computational considerations, when learning parameters, several fundamental mathematical questions must be carefully considered. 1) First, the model’s dynamical range should be analyzed in order to ascertain whether there are nonidentifiable regimes. Dynamical systems may gravitate toward attractors and steady equilibria which are independent of certain model parameters and thus cannot be used to train the net. 2) Numerical stability must be maintained throughout the training process, e.g., by rescaling the neural net output to prevent numerical overflow. In certain parameter regimes, dynamical systems may also exhibit chaotic behavior that inhibits the learning process. The neural net must be kept from straying into the “danger zones” of potential numerical instability as it traverses the parameter space during training. 3) To speed up computation, a suitable neural net architecture should be chosen that encapsulates as much information on the parameters as can reasonably be assumed a priori. For instance, using activation functions that guarantee that parameters remain in valid ranges may be conducive: If parameters are probabilities, an activation function that maps into [0, 1] (e.g., a sigmoid) may be an appropriate choice for the final layer.

## Application to Time Series Data: Diffusive SIR Model of Epidemics

We first demonstrate our methodology by applying it to time series data generated by a classical agent-based model of epidemics. Consider *N* agents moving around a square domain [0, *L*]^2^ with periodic boundary conditions, 0 <  *L* ∈ ℝ; each agent has a position **x**_*i*_, and a state *k*_*i*_ ∈ {S, I, R}. All agents with *k*_*i*_ = S are susceptible to the disease. If a susceptible agent lies within the infection radius *r* of an infected agent (an agent with *k*_*i*_ = I), they are infected with infection probability *p*. After a certain recovery time *τ*, agents recover from the disease (upon which *k*_*i*_ = R); each agent’s time since infection is stored in a state *τ*_*i*_. Agents move randomly around the space with diffusivities *σ*_*S*_, *σ*_*I*_, and *σ*_*R*_. Each iteration of the agent-based model thus consists of the following steps: 


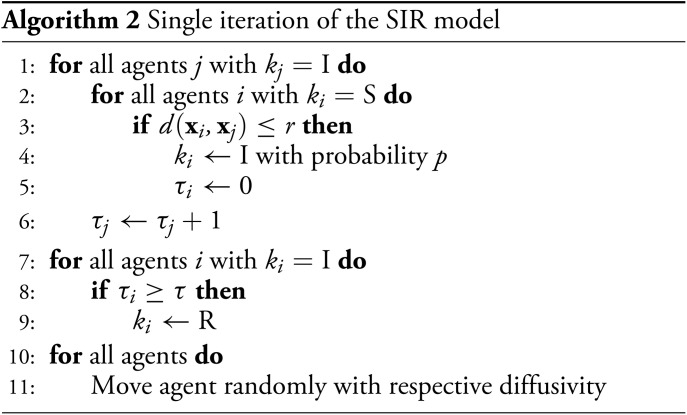


**Fig. 2.**
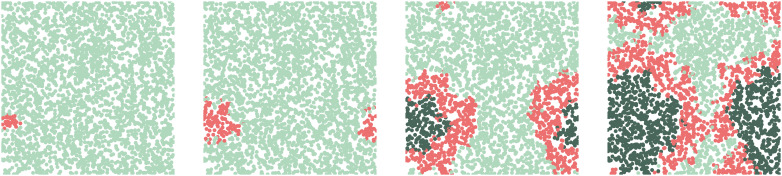
Diffusion of infection (red) through the agent population on a two-dimensional domain with periodic boundary conditions. Dark green: Recovered agents.

The function *d* is the distance metric on the torus

$$\begin{array}{c} d{\left(x,y\right)}^{2}=\sum_{i}\min{\left(|x_{i}-y_{i}\left|,L-\right|x_{i}-y_{i}|\right)}^{2}, \end{array}$$

and we initialize the ABM with a single infected agent at a random location see fig. 2. We set the infection time to *τ* = 14, the infection probability to *p* = 0.2, and the infection radius to *r* = 0.3. The space has dimension *L* = 10, and the generated time series contains 100 time steps.

Let *S*(**x**, *t*) be the spatiotemporal distribution of susceptible agents (analogously *I* and *R*). Assume that we observe only the temporal densities

$$\begin{array}{c} \;\text{S}\left(t\right)=\frac{1}{N}\int_{\Omega }S\left(x,t\right)dx, \end{array}$$

applicable to the spread of an epidemic, where we only see the counts of infected and recovered patients without any location tracking or contact tracing. To these observations, we now wish to fit the stochastic equations

$$\begin{array}{c} \begin{array}{c} d\;\text{S}=-\beta \text{SI}dt-\sigma \;\text{I}\circ dW,\\ d\;\text{I}=(\beta \;\text{S}-\tau^{-1})\;\text{I}dt+\sigma \;\text{I}\circ dW,\\ d\;\text{R}=\tau^{-1}\;\text{I}dt, \end{array} \end{array}$$

where *W* is a Wiener process and ∘ represents the Stratonovich integral. We will do so by recovering the parameters ***λ*** = (*β*, *τ*, *σ*)∈ℝ_+_^3^ from observations of S, I, and R generated by the agent-based model. We expect *β* ≈ *p*, as the likelihood of a susceptible agent coming in contact with an infected agent approaches 1. Naturally, there is some data–model mismatch, which is intentional and in this case accounted for by the noise *σ*; in reality, *σ* could for instance model errors in the estimates of the number of infected agents.

We use a shallow neural net with 20 neurons in the hidden layer and, since parameters are known to be positive and negative outputs would produce unpredictable behavior, the modulus *x* ↦ |*x*| as an activation function. The loss function is the batch-averaged mean squared error,

[5] $$\begin{array}{c} J\left({\hat{\varphi }}_{i},...,{\hat{\varphi }}_{i+B},\varphi_{i},...,\varphi_{i+B}\right)=\frac{1}{B}\sum_{j=i}^{i+B}{\left\|{\hat{\varphi }}_{j}-\varphi_{j}\right\|}^{2}, \end{array}$$

and we use the Adam optimizer (50) for the backpropagation. For a single initialization, we train the neural net for 70 epochs with a batch size of 90, and we run the model from 20 different initializations.

**Fig. 3.**
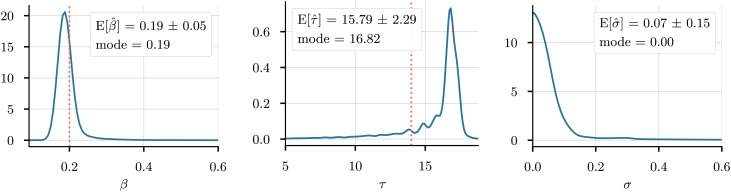
Marginal densities for the parameters ***λ*** = (*β*, *τ*, *σ*), calculated using Eq. **4**, smoothed with a Gaussian kernel. The parameters used to run the agent-based model are *p* = 0.2 and *τ* = 14 (indicated by the red dotted lines).

**Fig. 4.**
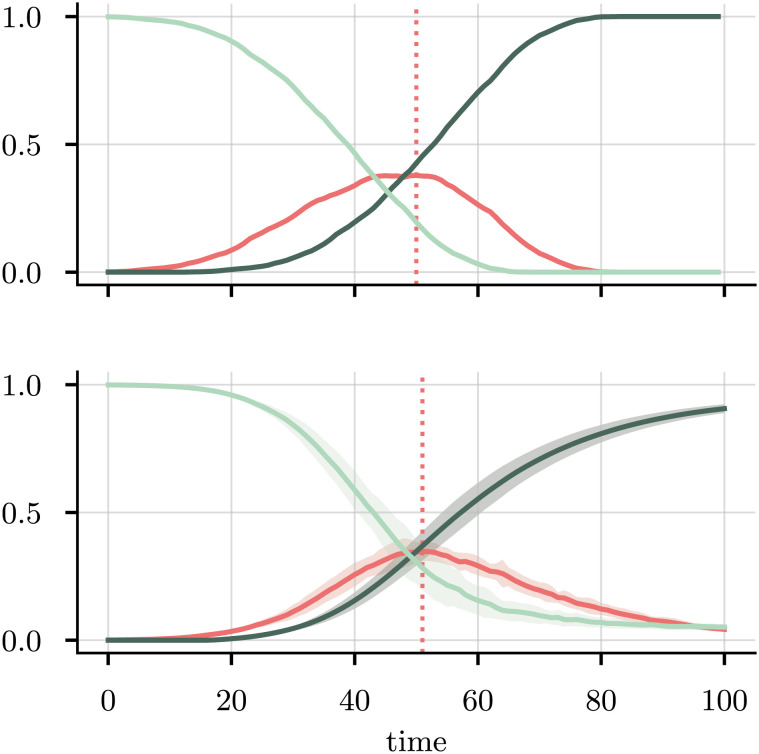
SIR densities S(*t*) (light green), I(*t*) (red), and R(*t*). *Top*: Sample densities used to train the neural net, generated by running the agent-based model with *N* = 3,000 agents for 100 iterations. *Bottom*: Predicted densities from the calibrated model, using the neural net outputs after training, averaged over 20 different initializations, with the error bands showing one SD. For each initialization, the neural net is trained for 70 epochs, with a batch size of 90. Dotted lines: Times of peak infection. Total runtime: 44.8 s.

Fig. 3 shows the parameter predictions. We recover the infection probability with a maximum likelihood estimate of $\hat{\beta }=0.19$ and a slightly overestimated infection time $\hat{\tau }=16.82$. The most likely noise level is predicted to be 0, with an expectation value of 0.07 ± 0.15. In Fig. 4, we use the neural net predictions from all 20 initializations to calibrate the model. We see how, on average, the predicted time of peak infection matches the true time (dotted lines), though the density of infected agents both increases and decreases significantly more slowly than in the true dataset. Despite the significant data–model mismatch, the neural network therefore manages to make reasonable predictions—both in terms of the estimated parameters and the output of the calibrated ABM—in only a few seconds: Training the model for all 20 initializations took 45 s on a standard laptop CPU.

## Application to a Nonconvex Problem: the Harris–Wilson Model of Economic Activity

In this section, we analyze the connection between prediction uncertainty and noise in the training data and compare the method to more classical methods with regard to its predictive ability and computational performance. We do so using the Harris–Wilson model of economic activity on a network (19), a nonconvex problem for which the loss function has at least two global minima. In a first step, we will consider synthetic data, thereby avoiding any data–model mismatch and giving us control over the variance in the data; thereafter, we shall analyze a real dataset of economic activity in Greater London. Before presenting our results, we briefly describe the model dynamics.

In the Harris–Wilson model, *N* origin zones are connected to *M* destination zones through a directed, weighted, complete bipartite network, i.e., each origin zone is connected to every destination zone. Economic demand flows from the origin zones to the destination zones, which supply the demand. Such a model is applicable for instance to an urban setting, the origin zones representing, e.g., residential areas, and the destination zones representing retail areas, shopping centers, or other areas of consumer activity. Let **C** ∈ ℝ^*N* × *M*^ be the nonzero section of the full network adjacency matrix. The network weights *c*_*ij*_ quantify the convenience of traveling from origin zone *i* to destination zone *j*: a low weight thus models a highly inconvenient route (e.g., due to a lack of public transport). Each origin zone has a fixed demand *O*_*i*_. The resulting cumulative demand at some destination zone *j* is given by

$$\begin{array}{c} D_{j}=\sum_{i=1}^{N}T_{\mathrm{ij}}, \end{array}$$

*T*_*ij*_ representing the flow of demand from *i* to *j*. The model assumption is that this flow depends both on the size *W*_*j*_ of the destination zone and the convenience of “getting from *i* to *j*”:

$$\begin{array}{c} T_{\mathrm{ij}}=\frac{W_{j}^{\alpha }c_{\mathrm{ij}}^{\beta }}{\sum_{k=1}^{M}W_{k}^{\alpha }c_{\mathrm{ik}}^{\beta }}O_{i}. \end{array}$$

The parameters *α* and *β* represent the relative importance of size and convenience to the flow of demand from *i* to *j*: High *α* means that consumers value large destination zones (e.g., prefer larger shopping centers to smaller ones) and high *β* means that consumers place a strong emphasis on convenient travel to destination zones. Finally, the sizes *W*_*j*_ are governed by a system of *M* coupled logistic equations:

[6] $$\begin{array}{c} dW_{j}=\epsilon W_{j}\left(D_{j}-\kappa W_{j}\right)dt+\sigma W_{j}\circ dB_{j}, \end{array}$$

with given initial conditions *W*_*j*_(*t* = 0)=*W*_*j*, 0_. Here, *ϵ* is a responsiveness parameter, representing the rate at which destination zones can adapt to fluctuations in demand, and *κ* models the cost of maintaining a larger site per unit floor space (e.g., rent, utilities, etc.). We recognize the logistic nature of the equations: The change in size is proportional to the size itself, as well as to *D*_*j*_ − *κW*_*j*_. A low value of *κ* favors larger destination zones (e.g., larger malls), a high cost favors smaller zones (e.g., local stores). In addition, the model Eq. **6** includes multiplicative noise with variance *σ* ≥ 0, with ∘ signifying Stratonovich integration. This represents a perturbation of the net capacity term *D*_*j*_ − *κW*_*j*_ by a centered Gaussian. We thus have a system of *M* coupled stochastic differential equations. In the noiseless case, the stable equilibrium is determined by

[7] $$\begin{array}{c} W=\kappa^{-1}D, \end{array}$$

where **W** ∈ ℝ^*M*^ and **D** ∈ ℝ^*M*^ are the origin zone sizes and demands, respectively. The steady state is thus independent of the responsiveness *ϵ*, which affects only the convergence rate to the equilibrium. We can therefore set *ϵ* = 1. Note also that *α* and *β* are unitless and thus unaffected by any scaling of the origin or destination zone sizes. *κ* is given in units of cost/area, and scales accordingly.

In the stochastic case, the dynamics reach a steady-state equilibrium that is independent of the initial condition **W**_0_. A good indicator to assess the effect of the parameters *α* and *β* on the system steady state is the inequality

[8] $$\begin{array}{c} \nu :=\underset{i,j}{\sup}\frac{W_{j}-W_{i}}{\sum_{k}W_{k}}\in \left[0,1\right]; \end{array}$$

**Fig. 5.**
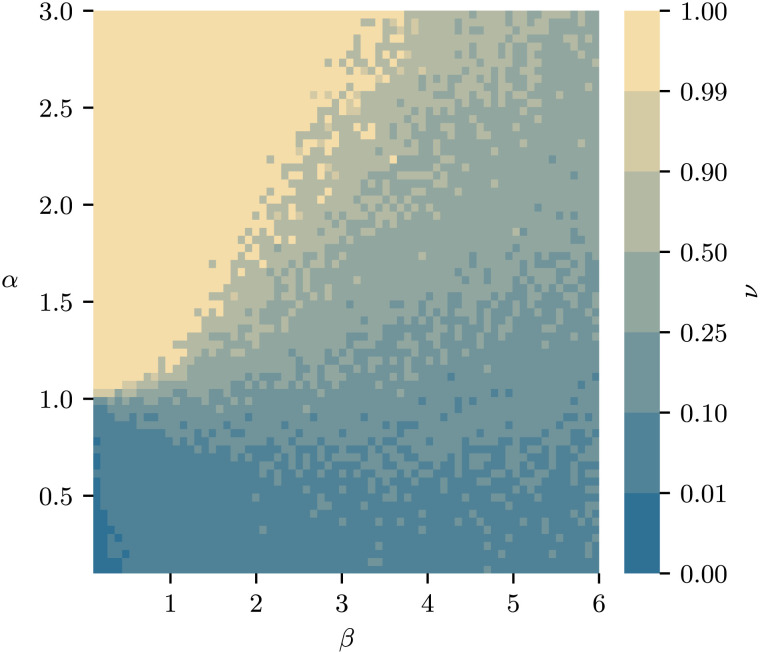
The inequality parameter *ν* (Eq. **8**) of the destination zone sizes as a function of *α* and *β* (holding *κ* fixed). For high *β* and low *α*, a larger number of smaller centers can emerge, whereas for high *α* and relatively low *β*, a small number of supercenters can form, until finally the market becomes entirely dominated by a single zone.

*ν* = 1 indicates a completely monopolized market, while *ν* = 0 indicates perfect equality of size, i.e., all zones are of equal size. Low values of *α* and high values of *β* (i.e., low relative importance of zone size and high relative importance of convenience) lead to low overall inequality, due to a large collection of smaller zones emerging, all of roughly equal size (cf. Fig. 5). Conversely, low relative importance of travel convenience and high relative importance of store size lead to the emergence of a small number of very large superstores, with most smaller centers dying out: This is reflected in the high values of *ν*.

The steady-state condition Eq. **7** is given by the *M* coupled equations

[9] $$\begin{array}{c} \sum_{i}\frac{c_{\mathrm{ij}}^{\beta }O_{i}}{\sum_{k}W_{k}^{\alpha }c_{\mathrm{ik}}^{\beta }}=\kappa W_{j}^{1-\alpha },\;j=1...M. \end{array}$$

In the case of *ν* = 1, Eq. **9** will be solved by any $\hat{\alpha }$ and $\hat{\beta }$, with $\hat{\kappa }$ uniquely given by

$$\begin{array}{c} \hat{\kappa }=\frac{\sum_{i}O_{i}}{W_{\hat{k}}}, \end{array}$$

where $\hat{k}$ is the index of the monopolizing zone. This region is thus unlearnable in *α* and *β* (with consumers having only a single option, both their preference for size and convenience is irrelevant). Similarly, the case *ν* = 0 admits solutions that are independent of *α* (since all zones are of the same size, the size preference parameter becomes irrelevant). Parameters thus cannot be learned for *ν* ∈ {0, 1}, and throughout this work, we only consider datasets with 0 <  *ν* <  1.

For any *ν*, the triple (*α* = 1, *β* = 0, *κ* = ∑_*i*_*O*_*i*_/∑_*k*_*W*_*k*_) represents a global minimum of the loss function, and if 0 <  *ν* <  1, it can be shown that there exist at most two global minima of the loss function (see ref. 19 for a proof):

**Fact.** *In their stable equilibrium, the noiseless Harris–Wilson equations Eq. **7** for 0 <  *ν* <  1 admit at most two solutions in (*α*, *β*, *κ*), one being the trivial solution (1, 0, ∑_*i*_*O*_*i*_/∑_*k*_*W*_*k*_). For any *ν*, the solution is unique in *κ*, where *κ* is given by ∑_*i*_*O*_*i*_/∑_*k*_*W*_*k*_.*

### Results on Synthetic Data.

We generate synthetic data of the steady-state sizes **W**^⋆^ := **W**(*t* → ∞), determined via sup_*j*_d*W*_*j*_ <  tol for some sufficiently small tolerance. We generate training data with different noise levels; the data have length *L* = 1 in the noiseless and *L* = 4 in the noisy case. We run the numerical solver without noise since we do not wish to include any assumptions about *σ* in the training process.

**Fig. 6.**
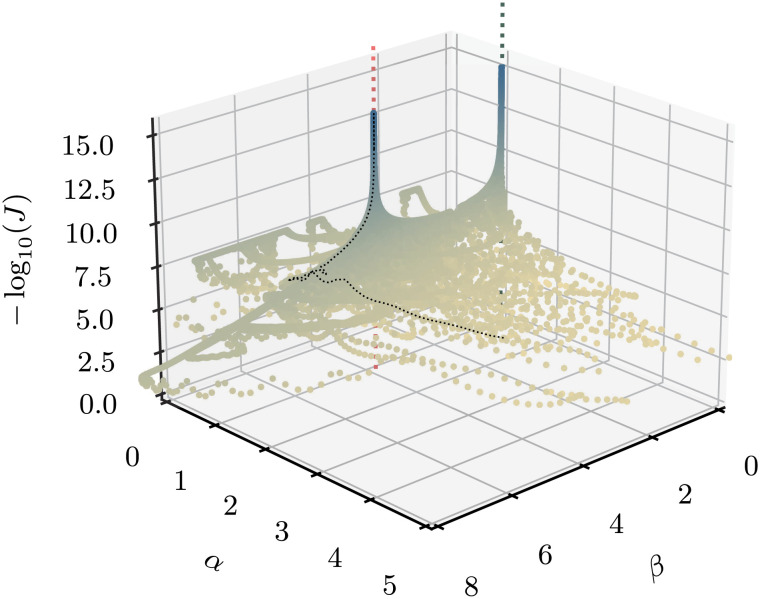
The negative logarithm of the loss potential *J* as a function of the estimates $\hat{\alpha }$ and $\hat{\beta }$. Each point represents a single training step, and the model was trained for 10,000 iterations over 100 different initializations. The two minima of the potential at the true parameters (*α* = 1.2, *β* = 4, *κ* = 2, red line) and the trivial solution (*α* = 1, *β* = 0, *κ* = 2, green line) are clearly visible. Also shown is an example trajectory the model takes as it converges to the (nontrivial) solution (black dotted line).

Considering first the noiseless case, we wish to estimate a probability distribution for the model parameters {*α*, *β*, *κ*}∈ℝ_+_^3^ with confidence bounds (*ϵ* is not learnable, as it does not affect the steady state). The steady state must meet the criterion 0 <  *ν* <  1. We train the neural net 100 times from different initializations for 10,000 epochs each, performing a gradient descent step after each prediction (thus, the batch size is *B* = 1) and using the same network architecture as before. The loss function is the same as in the SIR case (Eq. **5**). Fig. 6 visualizes the resulting loss potential in *α* and *β*. We clearly see the two global minima of *J*, one at the true parameters and one at the trivial minimum of the decoupled system *α* = 1, *β* = 0. In running the model several times with different initializations, we are thus able to deal with the nonconvexity of the problem.

**Fig. 7.**
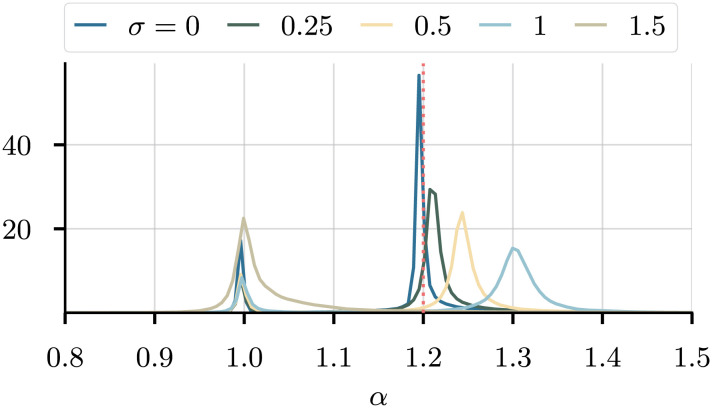
The marginal densities for *α* as a function of the noise in the data, smoothed with a Gaussian kernel. As we increase the noise, the peak width increases (Fig. 8). Red dotted line: True value. Similar results hold for the other parameters; *SI Appendix*, Fig. S3.

**Fig. 8.**
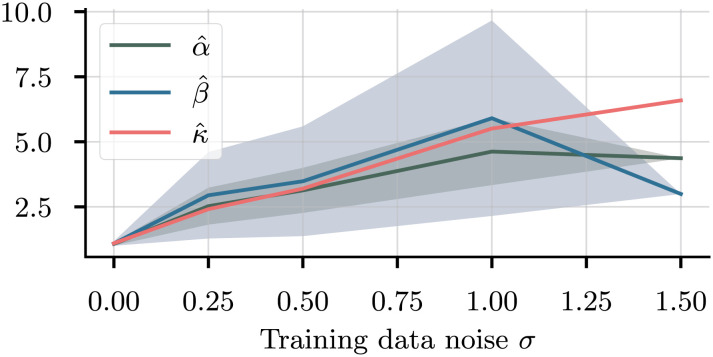
Average peak width (defined as the width at half height) for the three parameters. Shaded area: Standard deviation. For very high noise, only the trivial peak is found, wherefore the standard deviation becomes 0.

Turning to the case of noisy data, Fig. 7 shows the marginals for *α* as we increase the level of noise in the data. The noise is generated by a mean-zero, centered Gaussian, and its variance ranges from 0 to a very high level of *σ* = 1.5. In the noiseless case (dark blue), the nontrivial solution dominates, with a small peak at the trivial case *α* = 1. As the noise increases, so do the peak widths, until at *σ* = 1.5 the nontrivial solution is no longer identifiable, and the model collapses to the trivial value *α* = 1. Fig. 8 shows the average and SD of the peak widths for all three parameters. Since *κ* is uniquely identifiable, there is only one peak; hence, the SD is 0. For the other two, the disappearance of the nontrivial peak is evident at *σ* = 1.5, where the SD of the peak width becomes 0. For all three parameters, we observe an increase in the average peak width as the noise in the data increases. At *σ* = 0, there is still some uncertainty in the predictions, originating from the uncertainty in the internal model weights ***θ***.

Generating the 1 million samples in 3-dimensional space shown in Fig. 6 takes about 15 min on a standard CPU. Far fewer data points would already have been sufficient to get good estimates of the parameters since the model spends most of its time near the minima of the loss function. The time to run a single iteration increases with (*N* + *M*)^2^ as the adjacency matrix of the network (which is not sparse) grows accordingly; the loss after a fixed number of iterations remains fairly constant (*SI Appendix*, Fig. S2). In the following section, we give a more detailed analysis of the model performance by comparing it to that of a classical Bayesian estimator.

### Results on Data of Economic Activity in London.

We apply our methodology to a dataset of economic activity in Greater London and compare the results to a study of the same dataset, performed using an MCMC approach (20). The data consist of origin zone sizes **O**, destination zone sizes **W**, and two different convenience matrices **C** for the region for the period around 2015/2016.

#### Origin zones.

The origin zone sizes are given by the total spending budget of each of the *N* = 625 electoral wards in Greater London as classified by the Greater London Authority (GLA) (53). Ward-level income is calculated as the product of the number of households times the average household income; however, households do not spend their entire budget on retail and service goods: According to the Office for National Statistics (54), between 2015 and 2017 London households on average spent between 21% and 30% of their total budget on comparison, service, and convenience goods^*^; hence, we multiply the income figures by 0.21 to obtain the origin size (we are excluding smaller retail zones from the dataset and hence choose to exclude food costs from the budget figure, see footnote). For example, for the City of London, 6680 households and an average household income of £63.620/a result in a total ward-level income of about £425 million per annum and hence an origin size of £89 million/annum. In order to prevent numerical overflow, we use units of £10^8^/a for the origin zones. Recall that scaling the origin zone values only affects the resulting prediction for *κ*, as *α* and *β* are unitless. It should be noted that while the population figures are for 2015, the income figures are only for 2012/2013.

#### Destination zones.

The destination zone sizes are the total occupied retail floor space sizes in m^2^ for all *M* = 49 town centers classified as either “international,” “metropolitan,” or “major” by the Greater London Authority (GLA) (55). In the GLA report (55), this retail floor space comprises comparison, convenience, and service retail (as given by ref. 55, Table 1.1.1 A–C); for example, for the West End, we obtain a total occupied retail floor space in 2016 of 474.456 m^2^. In order to prevent numerical overflow (resulting from large values of *W*_*j*_^*α*^ potentially occurring during the training), we use units of 10^5^m^2^ for the destination zone sizes. With this choice of units for the origin and destination zone sizes, *κ* will be given in units of £1000/a. The true value resulting from this data is *κ* = ∑_*i*_*O*_*i*_/∑_*k*_*W*_*k*_=£8301/a.

**Table 1.** Comparison of predicted values, expected calibration MSPE, and compute times

Noise regime		Temporal metric	Spatial metric	Euclidean metric (MCMC)
*σ* = 0.014 (low noise)	Predictions $\left(\hat{\alpha },\hat{\beta },\hat{\kappa }\right)$	(0.99, 0.02, 8.26)	(0.99, 0.02, 8.25)	(1.18, 0.28, (8.3))
	Expected MSPE	(1.7 ± 1.2)×10^−8^	(1.6 ± 1.1)×10^−8^	(4.5 ± 0.1)×10^−5^
	compute time	1 min 8 s	1 min 8 s	3 h 40 min
*σ* = 0.14 (high noise)	Predictions $\left(\hat{\alpha },\hat{\beta },\hat{\kappa }\right)$	(0.92, 0.54, 7.45)	(0.92, 0.39, 7.39)	(0.12, 0.75, (8.3))
	Expected MSPE	(1.8 ± 1.3)×10^−6^	(1.7 ± 1.1)×10^−6^	(2.3 ± 0.2)×10^−4^
	compute time	1 min 8 s	1 min 8 s	7 h 20 min

#### Cost network.

For the cost network **C**, we use the Google Distance Matrix API^†^ to extract travel data from Google Maps. The API can be used to extract both travel distances and travel times for large batches of search queries for all the transport modes available on Google Maps (driving, public transport, walking, and cycling). We consider only two modes of transport: driving and public transport. The API does not provide past data, so travel time and distance data reflect the state of the network in June 2022. In order to restore some level of comparability, we set the travel date to a Sunday in order to blend out the newly opened Elizabeth line (which in June 2022 did not yet run on Sundays). We define the distance *d*_*ij*_ between two nodes as the shorter of the two transport modes considered: For example, traveling from Kentish Town to the West End takes about 19.7 min by public transport and 22.35 min by car, so we set the distance to be 19.7 minutes (of course, this assumes that everyone can choose between both modes of transport and does not factor in the added cost of driving in London). Note that the Google Maps public transport mode defaults to walking for very short distances. We consider two different metrics: a temporal metric and a spatial metric. The convenience factors are derived in ref. 56 as

**Fig. 9.**
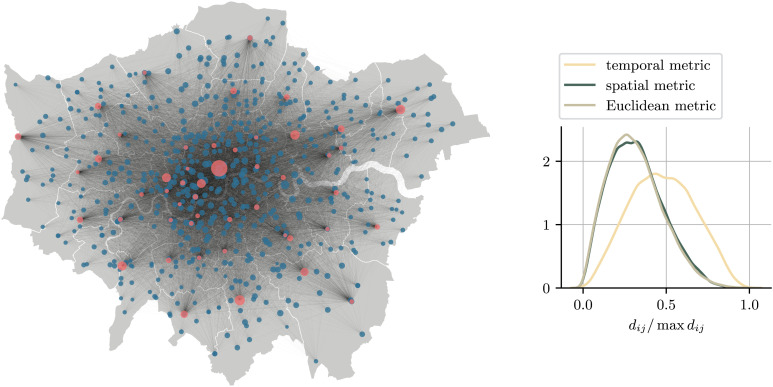
*Left*: Visualization of the London dataset, as described in the text. Blue dots are the origin zone sizes for all *N* = 625 wards; red dots are retail floor space sizes for *M* = 49 retail centers. The network edge weights are given by Eq. **10**, using the minimum transport time between locations for public transport or driving. Background map of London boroughs: (52). *Right*: The densities of the distance distributions for three different metrics: temporal, spatial, and Euclidean.

[10] $$\begin{array}{c} c_{\mathrm{ij}}=e^{-d_{\mathrm{ij}}/\tau }, \end{array}$$

where *d*_*ij*_ is the distance in the metric in question and *τ* = sup_*i*, *j*_*d*_*ij*_ the time/length scale, ensuring a unitless exponent. The network is then given by **C** = (*c*_*ij*_). Fig. 9 visualizes the dataset: The red nodes are the destination zones *W*_*j*_, the blue dots are the origin zones *O*_*i*_, and the network edge widths represent the cost matrix, using the temporal metric. The large, central destination zone is the London West End, by far the largest retail zone in London. On the right, the distance distributions for the two metrics are shown: It is interesting to observe that the temporal metric has a distribution more or less symmetrically centered around 0.5, whereas the distance metric is much more heavily skewed to the left. In practice, this means that zones are statistically closer spatially than temporally. This may be an artifact of our choice of transportation mode: Since users must use either public transport or drive, zones within realistic walking or cycling distance appear further away than they actually are. This is substantiated by the fact that more zones lie within zero spatial distance of each other than within zero temporal distance. We expect the choice of metric to affect only the predictions for *β*, the convenience parameter coupling the dynamics to the network.

## Results

**Fig. 10.**
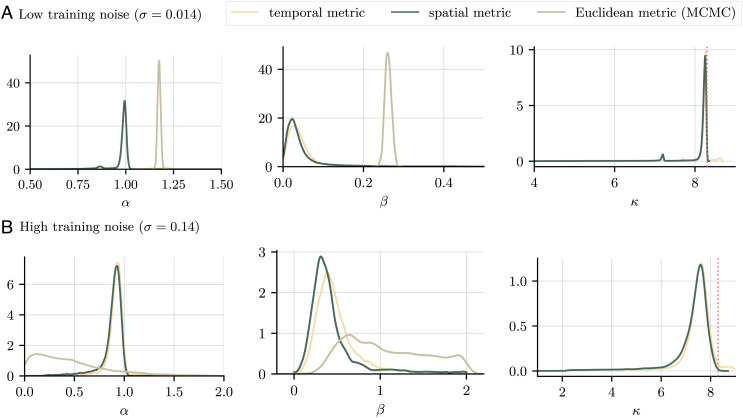
Marginal densities for ***λ*** = (*α*, *β*, *κ*) for different network metrics and two noise regimes, a low noise regime (*A*), and a high noise regime (*B*). Densities are obtained by running the model from 20 different initial seeds for 10,000 epochs each and smoothed with a Gaussian kernel. The true value of *κ* is given by the red dotted line. Runtime for each metric and noise regime: 1 min 8 s. Also shown are the densities for *α* and *β* obtained by running the MCMC analysis presented in ref. 20 (light brown). Runtime: 3 h 40 min (low noise), 7 h 20 min (high noise).

We estimate the marginal densities for the parameters for both metrics (temporal and spatial) and compare the results to those obtained in ref. 20. In that study, the authors considered two underlying noise levels in the data, $\sigma =\sqrt{2}\times 10^{-2}$ and $\sigma =\sqrt{2}\times 10^{-1}$, and estimated densities for *α* and *β*. We produce estimates for *α*, *β*, and *κ*, using the same values for the noise in the forward pass of the scheme. The resulting marginals are shown in Fig. 10, along with the densities obtained from running the MCMC scheme from ref. 20. We train our model for 10.000 epochs over 20 different initializations to obtain the same number of samples. It should be noted that the authors in ref. 20 used Euclidean distances for their study, which however in Fig. 10 can be seen to have much the same distribution as our spatial metric.

Table 1 summarizes the results. While the MCMC scheme produced very different estimates depending on the choice of *σ*, our method shows good consistency across noise regimes. As is to be expected, the predictions are independent of the choice of metric for *α* and *κ*. We obtain good to fair estimates for *κ* as well as a consistent maximum likelihood estimate for *α*. We assess the quality of the predictions by using the most likely values to calibrate the numerical solver Eq. **6** and computing the expected mean squared prediction error over 1,000 runs (thereby accounting for the random noise involved). Since (20) did not estimate *κ*, we use its true value for the calibration run with the MCMC values. As can be seen, our method improves on the MCMC prediction by three orders of magnitude in the low noise and two orders of magnitude in the high noise regime. Note also that our method produces density estimates in just over one minute, while the MCMC scheme took between 3.7 h and 7.3 h to run; this represents a performance improvement of two orders of magnitude. The speed-up is not attributable to parallelization: in CPU time, our method took 9 min to run, still representing two orders of magnitude performance increase.

## Conclusions

In this article, we outlined an approach to estimating probability densities for parameters of differential equations using neural nets. We considered a broad spectrum of datasets, including time series data of different lengths, steady-state data with only a single time frame, noiseless and noisy data, synthetically generated, and real data; the method was applied to a nonconvex problem with two global minima of the loss function (Harris–Wilson model), as well as to a situation with data–model mismatch (SIR model). In all cases, the neural net quickly and reliably found densities for the relevant parameters. We assessed the quality of the predictions by comparing the parameter predictions to their true values, in the case of the Harris–Wilson model, comparing our model’s prediction errors to that of a study using classical MCMC techniques. Our model significantly outperformed that study in terms of the accuracy of the predicted data, while being computationally much faster to run: In both regards, improvements covered two to three orders of magnitude. The use of numerical solvers for the forward pass allows giving estimates for different levels of assumed noise in the data, which can help to obtain realistic confidence bounds on predictions. However, in the SIR study, we also showed the ability of our method to learn the noise level itself. We demonstrated the importance of 1) analyzing a model’s dynamical range before training, 2) ensuring numerical stability throughout the training process, and 3) adapting the neural net architecture to the problem. For the Harris–Wilson model, this was achieved by 1) only training on data from the dynamical range 0 <  *ν* <  1, 2) scaling the origin and destination zone sizes appropriately to prevent numerical overflow, and 3) ensuring positive predictions by using the modulus as the activation function.

Due to the method’s performance and relative mathematical simplicity, we hope that it will prove a powerful tool across the quantitative sciences, such as in quantitative sociology or computational epidemiology. Our work opens up fruitful lines for further research: In particular, we have not yet considered how the model performance scales with the size of ***λ***. How, for instance, will the model fare when predicting very large parameter sets, such as graph adjacency matrices? This will be the subject of further investigation by the authors.

## Supplementary Material

Appendix 01 (PDF)

## Data Availability

All code and data can be found under https://github.com/ThGaskin/NeuralABM. It is easily adaptable to new models and ideas. The code uses the utopya package^‡^ (57, 58) to handle simulation configuration and efficiently read, write, analyze, and evaluate data. This means that the model can be run by modifying simple and intuitive configuration files, without touching code. Multiple training runs and parameter sweeps are automatically parallelized. The neural core is implemented using pytorch^§^. All datasets used in this work, including the synthetic data, have been made available, together with the configuration files needed to reproduce the plots. Detailed instructions are provided in *SI Appendix* and the repository.

## Footnotes

^*^The 30% figure comprises food and nonalcoholic drinks, alcoholic drinks and tobacco, clothing and footwear, household goods and services, and miscellaneous goods and services; this figure drops to 21% when excluding food and nonalcoholic drinks. See ref. 54, Table A.33.

^†^https://developers.google.com/maps/documentation/distance-matrix.

## References

[1] R. Verity , Estimates of the severity of coronavirus disease 2019: A model-based analysis. Lancet Infect. Dis. **20**, 669–677 (2020). 32240634 10.1016/S1473-3099(20)30243-7 PMC7158570

[2] A. Hogan , “Report 33: Modelling the allocation and impact of a COVID-19 vaccine” (Tech. rep., Imperial College London, 2020).

[3] S. Flaxman , Estimating the effects of non-pharmaceutical interventions on COVID-19 in Europe. Nature **584**, 257–261 (2020). 32512579 10.1038/s41586-020-2405-7

[4] B. F. Maier , Germany’s current COVID-19 crisis is mainly driven by the unvaccinated (2021).

[5] I. C. F. Team, Modeling COVID-19 scenarios for the United States. Nat. Med. **27**, 94–105 (2020). 33097835 10.1038/s41591-020-1132-9 PMC7806509

[6] R. Bhavnani, K. Donnay, D. Miodownik, M. Mor, D. Helbing, Group segregation and urban violence. Am. J. Polit. Sci. **58**, 226–245 (2013).

[7] D. Helbing , Saving human lives: What complexity science and information systems can contribute. J. Stat. Phys. **158**, 735–781 (2014). 26074625 10.1007/s10955-014-1024-9 PMC4457089

[8] D. Helbing, P. Molnár, Social force model for pedestrian dynamics. Phys. Rev. E **51**, 4282–4286 (1995). 10.1103/physreve.51.4282 9963139

[9] Y. Kuramoto, “Self-entrainment of a population of coupled non-linear oscillators” in International Symposium on Mathematical Problems in Theoretical Physics, H. Araki, Ed. (Springer-Verlag, 1975), pp. 420–422.

[10] C. Castellano, S. Fortunato, V. Loreto, Statistical physics of social dynamics. Rev. Mod. Phys. **81**, 591–646 (2009).

[11] M. D. Vicario , The spreading of misinformation online. Proc. Natl. Acad. Sci. U.S.A. **113**, 554–559 (2016). 26729863 10.1073/pnas.1517441113 PMC4725489

[12] J. B. Bak-Coleman , Combining interventions to reduce the spread of viral misinformation. Nat. Hum. Behav. **6**, 1372–1380 (2022). 35739250 10.1038/s41562-022-01388-6 PMC9584817

[13] E. F. Keller, L. A. Segel, Model for chemotaxis. J. Theor. Biol. **30**, 225–234 (1971). 4926701 10.1016/0022-5193(71)90050-6

[14] T. Vicsek, A. Czirók, E. Ben-Jacob, I. Cohen, O. Shochet, Novel type of phase transition in a system of self-driven particles. Phys. Rev. Lett. **75**, 1226–1229 (1995). 10060237 10.1103/PhysRevLett.75.1226

[15] F. Cucker, S. Smale, Emergent behavior in flocks. IEEE Trans. Automat. Control **52**, 852–862 (2007).

[16] R. Arditi, L. R. Ginzburg, Coupling in predator-prey dynamics: Ratio-Dependence. J. Theor. Biol. **139**, 311–326 (1989).

[17] S. Winkelmann, J. Zonker, C. Schütte, N. D. Conrad, Mathematical modeling of spatio-temporal population dynamics and application to epidemic spreading. Math. Biosci. **336**, 108619 (2021). 33887314 10.1016/j.mbs.2021.108619 PMC8054535

[18] K. Giesecke, G. Schwenkler, J. A. Sirignano, Inference for large financial systems. Math. Finance **30**, 3–46 (2020).

[19] B. Harris, A. G. Wilson, Equilibrium values and dynamics of attractiveness terms in production-constrained spatial-interaction models. Environ. Plan. A: Econ. Space **10**, 371–388 (1978).

[20] L. Ellam, M. Girolami, G. A. Pavliotis, A. Wilson, Stochastic modelling of urban structure. Proc. R. Soc. A: Math. Phys. Eng. Sci. **474**, 20170700 (2018). 10.1098/rspa.2017.0700 PMC5990696 29887748

[21] R. Rico-Martínez, K. Krischer, I. G. Kevrekidis, M. Kube, J. L. Hudson, Discrete- vs. continuous-time nonlinear signal processing of Cu electrodissolution data. Chem. Eng. Commun. **118**, 25–48 (1992).

[22] M. Ajelli , Comparing large-scale computational approaches to epidemic modeling: Agent-based versus structured metapopulation models. BMC Infect. Dis. **10**, 190 (2010). 20587041 10.1186/1471-2334-10-190 PMC2914769

[23] M. J. Lighthill, G. B. Whitham, On kinematic waves. II. A theory of traffic flow on long crowded roads. Proc. R. Soc. London. Ser. A, Math. Phys. Sci. **229**, 317–345 (1955).

[24] G. Toscani, Kinetic models of opinion formation. Commun. Math. Sci. **4**, 481–496 (2006).

[25] C. Wang, Q. Li, W. E, B. Chazelle, Noisy Hegselmann-Krause systems: Phase transition and the 2R-conjecture. J. Stat. Phys. **166**, 1209–1225 (2017).

[26] J. A. Carrillo, R. S. Gvalani, G. A. Pavliotis, A. Schlichting, Long-time behaviour and phase transitions for the Mckean–Vlasov equation on the torus. Arch. Ration. Mech. Anal. **235**, 635–690 (2019).

[27] R. A. Kasonga, Maximum likelihood theory for large interacting systems. SIAM J. Appl. Math. **50**, 865–875 (1990).

[28] G. A. Pavliotis, A. M. Stuart, Parameter estimation for multiscale diffusions. J. Stat. Phys. **127**, 741–781 (2007).

[29] X. Chen, Maximum likelihood estimation of potential energy in interacting particle systems from single-trajectory data. Elect. Commun. Proba. **26**, 45 (2021).

[30] L. Sharrock, N. Kantas, P. Parpas, G. A. Pavliotis, Parameter estimation for the Mckean-Vlasov stochastic differential equation. arXiv:2106.13751 [math.ST] (2021).

[31] M. Liu, H. Qiao, Parameter estimation of path-dependent McKean-Vlasov stochastic differential equations. Acta Math. Sci. **42**, 876–886 (2022).

[32] W. K. Hastings, Monte Carlo sampling methods using Markov chains and their applications. Biometrika **57**, 97–109 (1970).

[33] J. Kaipio, E. Somersalo, Statistical and Computational Inverse Problems (Springer Science and Business Media LLC, 2006), vol. **160**.

[34] A. M. Stuart, Inverse problems: A Bayesian perspective. Acta Numer. **19**, 451–559 (2010).

[35] A. Gelman , Bayesian Data Analysis (Chapman and Hall/CRC, 2013).

[36] G. A. Pavliotis, A. Zanoni, Eigenfunction martingale estimators for interacting particle systems and their mean field limit. arXiv:2112.04870 [math.NA] (2021).

[37] J. Ramsay, G. Hooker, Dynamic Data Analysis (Springer, New York, 2017).

[38] M. Timme, J. Casadiego, Revealing networks from dynamics: An introduction. J. Phys. A: Math. Theor. **47**, 343001 (2014).

[39] F. Lu, M. Maggioni, S. Tang, Learning interaction kernels in heterogeneous systems of agents from multiple trajectories. J. Mach. Learn. Res. **22**, 1–67 (2021).

[40] F. Lu, M. Maggioni, S. Tang, Learning interaction kernels in stochastic systems of interacting particles from multiple trajectories. Found. Comput. Math. **22**, 1013–1067 (2022).

[41] J. Wei , Emergent abilities of large language models. arXiv:2206.07682v2 [cs.CL] (2022).

[42] M. Raissi, P. Perdikaris, G. Karniadakis, Physics-informed neural networks: A deep learning framework for solving forward and inverse problems involving nonlinear partial differential equations. J. Comput. Phys. **378**, 686–707 (2019).

[43] E. Kharazmi, Z. Zhang, G. E. Karniadakis, hp-VPINNs: Variational physics-informed neural networks with domain decomposition. Comput. Methods Appl. Mech. Eng. **374**, 113547 (2021).

[44] S. Göttlich, C. Totzeck, Parameter calibration with stochastic gradient descent for interacting particle systems driven by neural networks. Math. Control Sig. Syst. **34**, 185–214 (2021).

[45] J. Dyer, P. Cannon, J. D. Farmer, S. Schmon, Black-box Bayesian inference for economic agent-based models, INET Oxford Working Paper No. 2022–05 (2022).

[46] J. Sirignano, K. Spiliopoulos, Mean field analysis of neural networks: A central limit theorem. arXiv:1808.09372 [math.PR] (2018).

[47] J. Sirignano, K. Spiliopoulos, Mean field analysis of neural networks: A law of large numbers. SIAM J. Appl. Math. **80**, 725–752 (2020).

[48] P. Kidger, On neural differential equations. arXiv:2202.02435 [cs.LG] (2022).

[49] P. Nakkiran , Deep double descent: Where bigger models and more data hurt. J. Stat. Mech.: Theory Exp. **2021**, 124003 (2021).

[50] D. P. Kingma, J. Ba, Adam: A method for stochastic optimization. arXiv:1412.6980 [cs.LG] (2014).

[51] Pytorch Documentation, Autograd mechanics (2022).

[52] Greater London Authority, Statistical GIS boundary files for London (2011).

[53] Greater London Authority, 2015 ward profiles and atlas (2015).

[54] Office for National Statistics, Household expenditure by countries and regions (2015–2017).

[55] Greater London Authority, 2017 Health Check Report (2017).

[56] A. Wilson, A statistical theory of spatial distribution models. Trans. Res. **1**, 253–269 (1967).

[57] L. Riedel, B. Herdeanu, H. Mack, Y. Sevinchan, J. Weninger, Utopia: A comprehensive and collaborative modeling framework for complex and evolving systems. J. Open Source Softw. **5**, 2165 (2020).

[58] Y. Sevinchan, B. Herdeanu, J. Traub, dantro: A python package for handling, transforming, and visualizing hierarchically structured data. J. Open Source Softw. **5**, 2316 (2020).

