# Supplementary material for: Neural parameter calibration for large-scale multiagent models
Source: Proc Natl Acad Sci U S A. 2023 Feb 10;120(7):e2216415120. doi: 10.1073/pnas.2216415120 (PMC9963791; doi:10.1073/pnas.2216415120)
Supplement: Supplementary file 1 — Appendix 01 (PDF) [file pnas.2216415120.sapp.pdf]

Supporting Information for

Neural parameter calibration for large-scale multi-agent models

Thomas Gaskin[a, 1], Grigorios A. Pavliotis[b], Mark Girolami[c, d], and Thomas Gaskin, Griogorios A. Pavliotis, Mark Girolami[2]

[a]Department of Applied Mathematics and Theoretical Physics, University of Cambridge, Cambridge CB3 0WA, United Kingdom; [b]Department of Mathematics, Imperial College London, London SW7 2AZ, United Kingdom; [c]Department of Engineering, University of Cambridge, Cambridge CB2 1PZ, United Kingdom; [d]The Alan Turing Institute, London NW1 2DB, United Kingdom

Thomas Gaskin.
E-mail: trg34@cam.ac.uk

This PDF file includes:

Supporting text
Figs. S1 to S3
SI References

Supporting Information Text

1. Methodology

A. Neural Networks: Notation and Terminology. A *neural network* is a sequence of length $L \geq 1$ of concatenated transformations. Each *layer* of the net consists of L_i *neurons*, connected through a sequence of *weight matrices* $\mathbf{W}_i \in \mathbb{R}^{L_{i+1} \times L_i}$. Each layer applies the transformation

$$\sigma_i(\mathbf{W}_i \mathbf{x} + \mathbf{b}_i)$$

to the input $\mathbf{x}$ from the previous layer, where $\mathbf{b}_i \in \mathbb{R}^{L_{i+1}}$ is the *bias* of the i-th layer. The function $\sigma_i : \mathbb{R}^{L_{i+1}} \to \mathbb{R}^{L_{i+1}}$ is the *activation function*; popular choices include the *rectified linear unit* (ReLU) activation function $\sigma(x) = \max(x, 0)$, and the *sigmoid activation function* $\sigma(x) = (1 + e^{-x})^{-1}$. A neural net has an *input layer*, an *output layer*, and *hidden layers*, which are the layers in between the in- and output layers. If a network only has one hidden layer, we call it *shallow*, else we call the neural net *deep*.

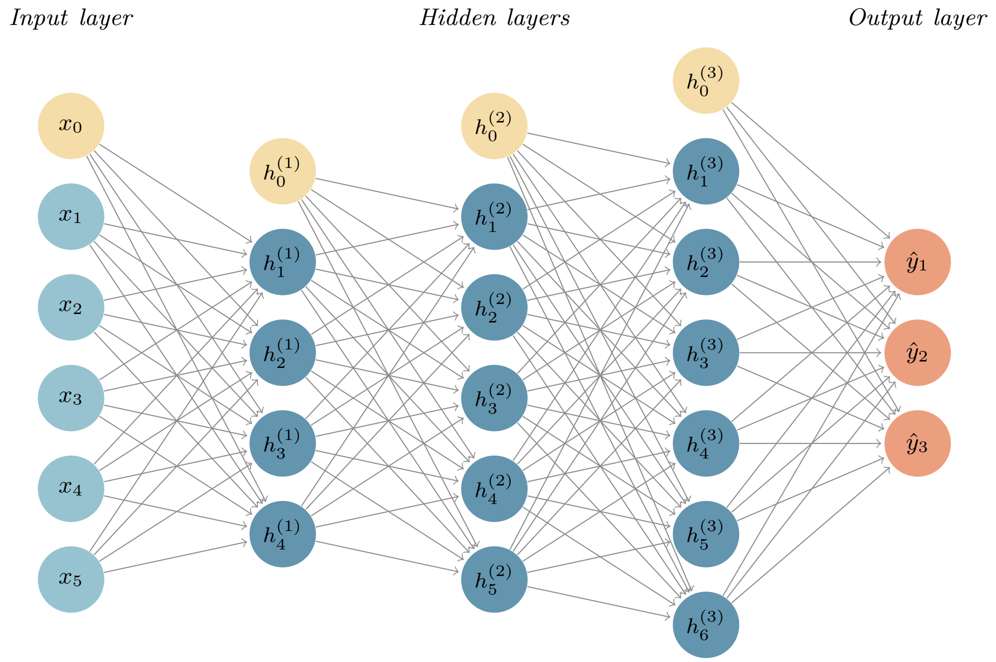

Fig. S1. Example of a deep neural network with 3 hidden layers. The inputs (light blue nodes) are passed through the layers, with links between layers representing the weight matrices $\mathbf{W}$. Each layer also applies a *bias* (yellow nodes), with the network finally producing an output (orange).

B. Details on the code. The code is uploaded to the Github repository as given in the main text.

B.1. Installation. Detailed installation instructions are given in the repository. First, clone the repository, install the `utopya` package and all the required additional components into a virtual environment, for example via `PyPi`. In particular, install `pytorch`. Enter the virtual environment. Then, from within the project folder, register the project:

```
utopya projects register .
```

You should get a positive response from the utopya CLI and your project should appear in the project list when calling:

```
utopya projects ls
```

Note that any changes to the project info file need to be communicated to utopya by calling the registration command anew. You will then have to additionally pass the `-exists-action overwrite` flag, because a project of that name already exists. See

```
utopya projects register --help
```

for more information. Finally, register the SIR model via

```
utopya models register from-manifest models/SIR/SIR_info.yml
```

(and the Harris-Wilson model accordingly).

B.2. Running the code. To run the code, execute the following command:

```
utopya run <model_name>
```

By default, this runs the model with the settings in the `<model_name>_cfg.yml` file. All data and the plots are written to an output directory, typically located in `~/utopya_output`. To run the model with different settings, create a `run_cfg.yml` file and pass it to the model like this:

```
utopya run <model_name> path/to/run_cfg.yml
```

This is recommended rather than changing the default settings, because the defaults are parameters that are known to work and you may wish to fall back on in the future.

Plots are generated using the plots specified in the `<model_name>_plots.yml` file. These too can be updated by creating a custom plot configuration, and running the model like this:

```
utopya run <model_name> path/to/run_cfg.yml --plots-cfg path/to/plot_cfg.yml
```

See the Utopia tutorial for more detailed instructions.

All the images in this article can be generated using so-called *configuration sets*, which are complete bundles of both run configurations and evaluation configurations. For example, to generate the four frames of the SIR model, you can call

```
utopya run SIR --cfg-set ABM_data
```

This will run and evaluate the `SIR` model with all the settings from the `SIR/cfgs/ABM_data/run.yml` and `eval.yml` configurations.

B.3. Parameter sweeps. Parameter sweeps are automatically parallelised by `utopya`, meaning simulation runs are always run concurrently whenever possible. The data is automatically stored and loaded into a data tree. To run a sweep, simply add a `!sweep` tag to the parameters you wish to sweep over, and specify the values, along with a default value to be used if no sweep is performed:

```yaml
param: !sweep
  default: 0
  values: [0, 1, 2, 3]
```

Then in the run configuration, add the following entry:

```yaml
perform_sweep: true
```

Alternatively, call the model with the flag `--run-mode sweep`. The configuration sets used in this work automatically run sweeps whenever needed, so no adjustment is needed to recreate the plots used in this work.

B.4. Initialising the neural net. The neural net is controlled from the `NeuralNet` entry of the configuration:

```yaml
NeuralNet:
  num_layers: 4
  nodes_per_layer:
    default: 20
    layer_specific:
      1: 10
      2: 15
  biases:                   # optional; if this entry is omitted no biases are used
    default: ~              # default is None (indicted by a tilde in YAML)
    layer_specific:
      0: default            # use pytorch default (xavier uniform)
      -1: [-1, 1]           # uniform initialisation on a custom interval
  activation_funcs:
    default: sigmoid
    layer_specific:         # optional
      1: tanh
```

```yaml
      3:
        name: HardTanh      # you can also pass a function that takes additional args and/or kwargs
        args:
          - -2  # min_value
          - +2  # max_value
  learning_rate: 0.001      # optional; default is 0.001
  optimizer: SGD            # optional; default is Adam
```

`num_layers` specifies the depth of the net; `nodes_per_layer` controls the architecture: provide a `default` size, and optionally any deviations from the default under `layer_specific`. The keys of the `layer_specific` entry should be indices of the layer in question. The optional `biases` entry determines whether or not biases are to be included in the architecture, and if so how to initialise them. A default and layer-specific values can again be passed. Setting an entry to `default` initialises the values using the pytorch default initialiser, a Xavier uniform initialisation. Passing a custom interval instead initialises the biases uniformly at random on that interval, and passing a tilde `~` (`None` in YAML) turns the bias off. `activation_funcs` is a dictionary specifying the activation functions on each layer, following the same logic as above. Any pytorch activation function is permissible. If a function requires additional arguments, these can be passed as in the example above. Lastly, the `optimizer` keyword takes any argument allowed in pytorch. The default optimizer is the Adam optimizer (1) with a learning rate of 0.001.

The neural net can be initialised from different initial values in the parameter space by changing the *random seed* in the configuration:

```yaml
seed: 42
```

Sweeping over different initialisations is achieved by sweeping over the seed, as described in the previous section.

B.5. Training the neural net. The `Training` entry of the configuration controls the training process:

```yaml
Training:
  batch_size: 2
  to_learn: [param1, param2]
  true_parameters:
    param3: 0.4
  loss_function:
    name: MSEloss
    # can pass additional args and kwargs here ...
```

You must specify which parameters to learn, and pass the true values for the others if you are not learning all parameters. Under the `loss_function` key you can specify the loss function to use, and pass any arguments or keyword arguments it may require using an `args` or `kwargs` key. You can use any available pytorch loss function.

2. Application to time series data: diffusive SIR model of epidemics

A. Neural Network Architecture. For this section, we choose a shallow neural net with 20 neurons in the hidden layer and the absolute value function as the activation function to ascertain parameter predictions are positive. We initialise the biases uniformly at random with values in the unit interval $[0, 1]$ to further push the neural network towards positive predictions. We use the Adam optimizer with a learning rate of 0.001. The corresponding configuration looks like this:

```yaml
SIR:
  NeuralNet:
    num_layers: 1
    nodes_per_layer:
      default: 20
    biases:
      default: [0, 1]  # Initialise biases on [0, 1]
    activation_funcs:
      default: linear
      layer_specific:
        -1: abs # Modulus activation func
    learning_rate: 0.002
    optimizer: Adam
  Training:
    batch_size: 90            # the time series has length 100
    to_learn: [p_infect, t_infectious, sigma]
    loss_function:
      name: MSELoss
```

B. Training. We train the model using the following operation: let $\boldsymbol{\varphi}(t) = (S(t), I(t), R(t))$ be the current state of the model; then in each iteration, we do

$$\boldsymbol{\varphi}(t+1) = \mathrm{ReLU}(\boldsymbol{\varphi}(t) + \mathrm{d}\boldsymbol{\varphi}(t)), \qquad [1]$$

where $\mathrm{d}\boldsymbol{\varphi}(t)$ is given by

$$\mathrm{d}\boldsymbol{\varphi}(t) = \begin{pmatrix} -\hat{\beta} & -\hat{w} \\ \hat{\beta} & \hat{w} - f(\gamma\hat{\tau}, t) \\ 0 & f(\gamma\hat{\tau}, t) \end{pmatrix} \begin{pmatrix} S(t)I(t) \\ I(t) \end{pmatrix} \qquad [2]$$

The ReLU function ensures that densities do not become negative. The estimated value $\hat{w}$ is given by $\hat{\sigma}X$, where $\hat{\sigma}$ is the neural net prediction for the noise, and $X \sim N(0, 0.1)$ a normally distributed random variable with variance 0.1. The function f is given by

$$f(s,t) = \frac{1}{s}\mathrm{sigmoid}(kt/s) \qquad [3]$$

with $k = 1000$. It approximates a step function, ensuring that recovery only begins after a certain period. γ is a scaling factor, designed to ensure the three estimated parameters are roughly of the same order of magnitude. This increases the speed of the neural net's convergence to a loss function minimum, as the parameters are closer together, but is not required to achieve reliable results. When writing the data, $\hat{\tau}$ is scaled back to its original dimension. We choose $\gamma = 10$.

C. Running the code. The training data for the ABM is provided in the `data/SIR` folder, and is provided in `hdf5` format. To train the neural net, simply run the following command (make sure you are in the project folder, otherwise change the path to the dataset to an absolute path):

```
utopya run SIR --cfg-set Predictions
```

This will load the ABM data and run the model 20 times from different initialisations, finally producing the plots from the manuscript. You can change the number of sweeps, and the initial seeds, by changing the `seed` entry in the `run.yml` configuration. By default, it looks like this:

```
seed: !sweep
  default: 0
  range: [20]
```

3. Application to a non-convex problem: the Harris-Wilson model of economic activity

A. Neural Network Architecture. The neural network architecture is the same as for the SIR section, using the same optimizer with the same learning rate of 0.002. We initialise the biases of the neural net in the interval $[0, 4]$:

```
HarrisWilson:
  NeuralNet:
    num_layers: 1
    nodes_per_layer:
      default: 20
    activation_funcs:
      default: linear
      layer_specific:
        -1: abs
    biases:
      default: [0, 4]
    learning_rate: 0.002
    optimizer: Adam
  Training:
    to_learn: [alpha, beta, kappa]
    batch_size: 1
    true_parameters:
      sigma: 0
    loss_function:
      name: MSELoss
```

In the noiseless case we set the training noise to 0, as we are not learning the noise; for the noisy runs, we also learn the noise.

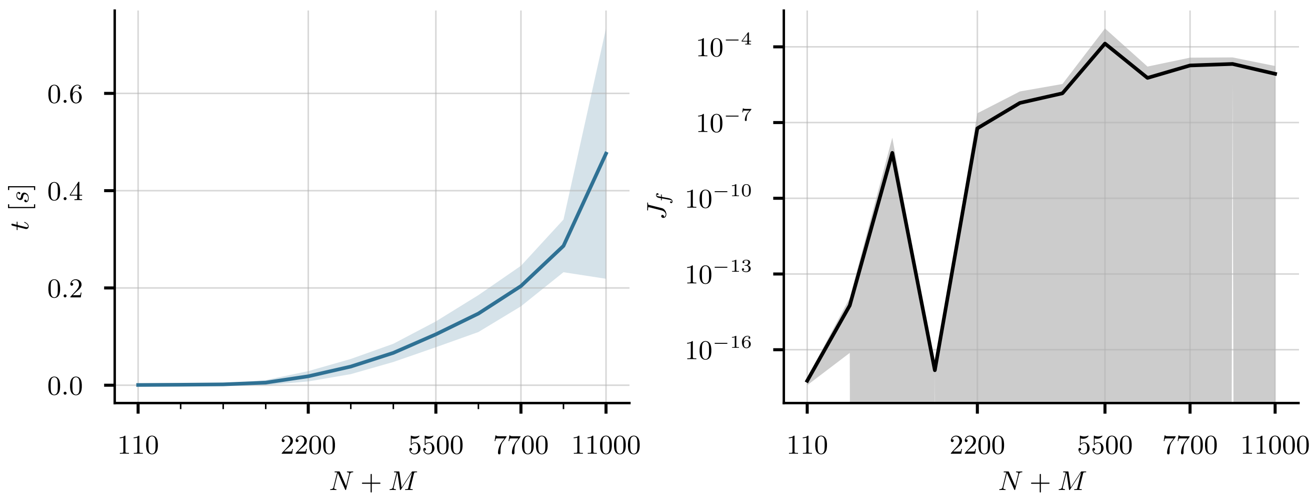

Fig. S2. Performance analysis of the model as a function of the network size $N + M$. Left: time to complete a single epoch ($L = 1$, $B = 1$). Right: training loss after 6000 epochs.

B. Training. We use the following matrix form of the Harris-Wilson equations to train the neural net. Let $\mathbf{D} \in \mathbb{R}^M$, $\mathbf{O} \in \mathbb{R}^N$, $\mathbf{W} \in \mathbb{R}^M$ be the demand vector, origin zone size vector, and destination zone size vector respectively. The the dynamics are given by

$$\mathbf{D} = \mathbf{W}^\alpha \odot \left[\left(\mathbf{C}^\beta \right)^T (\mathbf{O} \odot \mathbf{Z}) \right] \in \mathbb{R}^M, \tag{4}$$

with $\odot$ indicating the Hadamard product, and elementwise exponentiation. $\mathbf{Z} \in \mathbb{R}^N$ is the vector of normalisation constants

$$\mathbf{Z}^{-1} = \mathbf{C}^\beta \mathbf{W}^\alpha. \tag{5}$$

The dynamics then read

$$\dot{\mathbf{W}} = \mathbf{W} \odot \epsilon(\mathbf{D} - \kappa \mathbf{W}) \tag{6}$$

with given initial conditions $\mathbf{W}(t = 0) = \mathbf{W}_0$. This formulation is more conducive to machine learning purposes, since it contains easily differentiable matrix operations and does not use for-loop iteration.

C. Performance analysis. Figure S2 shows a performance analysis of the model as a function of the network size $N + M$. On the left, the time a neural net with 20 neurons takes to run a single epoch ($L = 1$, $B = 1$). Each data point is an average over 10 different initialisations, each with 6000 epochs. On the right, the loss after for each size after 6000 epochs is shown, averaged over 10 different initialisations. The shaded area represents the standard deviation. Training was performed on the CPU of a standard laptop.

D. Marginal densities. Figure S3 shows the corresponding marginals for β, κ, and the noise level σ for figure 7 in the main manuscript.

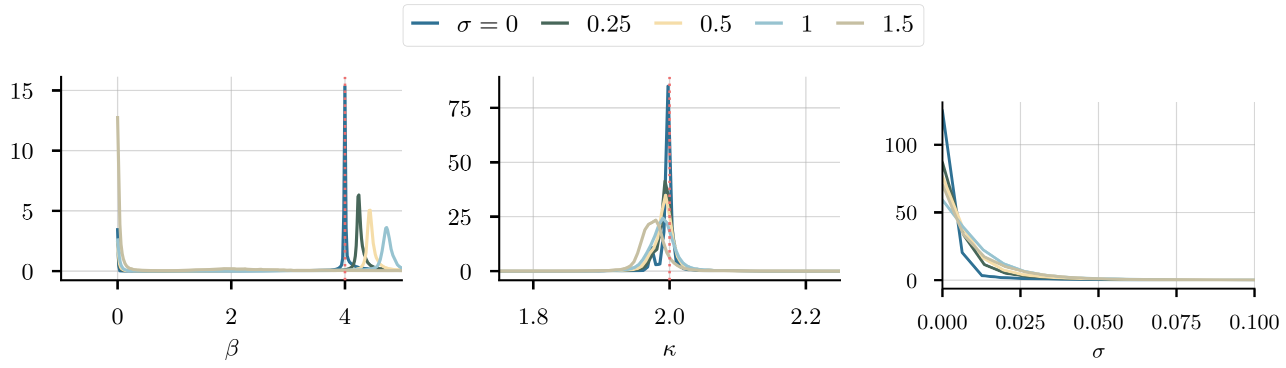

Fig. S3. Marginal densities for β, κ, and σ for different levels of noise in the training data, smoothed with a Gaussian kernel. All configurations as in figure 7 in the main manuscript. Red dotted lines: true values.

E. Datasets. The `data/HarrisWilson` folder contains several datasets, both real and synthetic, that can be used to train the neural net and learn parameters for the Harris-Wilson equations. Simply set the `load_from_dir` key in your `run.yml` file to point to the folder containing the data: the data will automatically be loaded and the model trained on that data.

E.1. Synthetic data. The `synthetic_data` folder contains synthetically generated networks, origin sizes, and destination sizes, both with and without noise. The name of the folder indicates the network size, i.e. `N_100_M_10` means $N = 100$ and $M = 10$. However, all folders also include a `config.yml` file detailing the specific configurations for each dataset.

E.2. London dataset. The `London_data` folder contains datasets of economic activity across Greater London. The `GLA_data` folder contains the data compiled from the two GLA studies on ward profiles and retail floor space. The `dest_sizes.csv` and `origin_sizes.csv` are the destination and origin zone sizes used in the paper. The `exp_times.csv` and `exp_distances.csv` are the two different transport network metrics used, calculated via $\exp(-d_{ij}/\max(d_{ij}))$ from the respective `distances.csv` and `times.csv` files. The `Google_Distance_Matrix_Data` folder contains transport times and distances using the Google Maps API service. Each file is a pickle-dictionary containing the API output for different travel modes: `transit` (public transport) and `driving` (driving, no traffic). The `departure_time` for transit is Sunday, June 19th 2022, 1 pm GMT (in Unix time: `departure_time = 1655640000`). However, since trips in the past cannot be computed, a future date must always be specified when using the API. The data is also available as a cost matrix in `.csv` format: entries are given in seconds and metres respectively.

F. Running the code. As with the SIR model, all the data (including the data from the GLA and Google Maps Distance API) is stored in the `data/HarrisWilson` folder. All data is given in `.csv` format. You can load data using the `load_from_dir` key:

```
HarrisWilson:
  Data:
    load_from_dir:
      network: data/London_data/exp_times.csv
      origin_zones: data/London_data/origin_sizes.csv
      destination_zones: data/London_data/dest_sizes.csv
```

Here, we are specifying the exact locations of the network, origin zone and destination zone size files. You can also pass a single directory to `load_from_dir`, as long as that directory contains `.csv` files labelled as `origin_sizes.csv`, `dest_sizes.csv`, and `network.csv`:

```
HarrisWilson:
  Data:
    load_from_dir: path/to/data
```

Run the model using the corresponding configuration sets to reproduce the plots; for example, to produce the plots of the London dataset, just do

```
utopya run HarrisWilson --cfg-set London_dataset
```

References

1. DP Kingma, J Ba, Adam: A method for stochastic optimization (2014).
